# Wideband nonlinear spectral broadening in ultra-short ultra - silicon rich nitride waveguides

**DOI:** 10.1038/srep27120

**Published:** 2016-06-08

**Authors:** Ju Won Choi, George F. R. Chen, D. K. T. Ng, Kelvin J. A. Ooi, Dawn T. H. Tan

**Affiliations:** 1 Photonics Devices and System Group, Engineering Product Development, Singapore University of Technology and Design, 8 Somapah Rd. 487372, Singapore; 2 Data Storage Institute, Agency for Science, Technology and Research (A*STAR), 2 Fusionopolis Way #08-01 Innovis, 138634, Singapore

## Abstract

CMOS-compatible nonlinear optics platforms with high Kerr nonlinearity facilitate the generation of broadband spectra based on self-phase modulation. Our ultra – silicon rich nitride (USRN) platform is designed to have a large nonlinear refractive index and low nonlinear losses at 1.55 μm for the facilitation of wideband spectral broadening. We investigate the ultrafast spectral characteristics of USRN waveguides with 1-mm-length, which have high nonlinear parameters (γ ∼ 550 W^−1^/m) and anomalous dispersion at 1.55 μm wavelength of input light. USRN add-drop ring resonators broaden output spectra by a factor of 2 compared with the bandwidth of input fs laser with the highest quality factors of 11000 and 15000. Two – fold self phase modulation induced spectral broadening is observed using waveguides only 430 μm in length, whereas a quadrupling of the output bandwidth is observed with USRN waveguides with a 1-mm-length. A broadening factor of around 3 per 1 mm length is achieved in the USRN waveguides, a value which is comparatively larger than many other CMOS-compatible platforms.

The development of complementary metal-oxide semiconductor (CMOS)-compatible platforms for nonlinear optics offers tremendous benefits to ultrafast all-optical signal processing and light generation^1^. In particular, nonlinear photonic chips with high Kerr nonlinearity enables low power self phase modulation (SPM), four wave mixing and cross phase modulation which collectively can give rise to the generation of ultrabroadband spectra via supercontinuum generation (SCG), which is applicable to fields including optical coherence tomography^2^, frequency metrology^3^, dense wavelength division multiplexed (DWDM) optical networks^4^ and so on.

Silicon-on-insulator (SOI) has been a popular platform due to high Kerr nonlinearity (typical nonlinear parameters of 100–300 W^−1^/m) and high refractive index contrast between Si core (*n*_*Si*_ = 3.48) and SiO_2_ cladding (*n*_*SiO*_*2*__ = 1.44) which facilitates tight confinement of light^1^. However, the nonlinear loss such as two photon absorption (TPA) and free carrier effect in telecommunication band with the wavelengths shorter than about 2000 nm limits the useable power levels and achievable nonlinear phase acquisition^5^,^6^. Chalcogenide glasses^7^,^8^,^9^,^10^ and AlGaAs^11^,^12^,^13^ are also promising platforms possessing high third-order nonlinearities, broadband transparency and low TPA, though limited to applications where CMOS compatibility is not required due to the challenging fabrication for highly efficient waveguides. In this way, CMOS-compatible devices based on these materials are in development.

Silicon nitride (Si_3_N_4_)^14^,^15^,^16^,^17^,^18^ and Hydex glass are two nonlinear optic platforms which have been used with much success to efficiently reduce nonlinear loss as well as linear loss. These two platforms yield increases in *n*_2_ according to Miller’s rule that is solely affected by linear refractive index^19^. The refractive index contrast between core and cladding is not as high as in SOI, so scattering losses from fabrication induced sidewall roughness can be made to be much smaller. Besides, TPA in silicon nitride and Hydex glass at the near-infrared can be also reduced owing to the large energy bandgap. Therefore, highly stable material and dispersion engineering properties have enabled these platforms to make significant progress within a short amount of time. In particular, frequency comb generations in Si_3_N_4_ and Hydex glass have been demonstrated with much success because of relatively higher nonlinear figure or merit than that in SOI^20^,^21^,^22^. However, the nonlinear refractive index is about ten times smaller than that of silicon. Another platform which is becoming more popular for nonlinear optics is silicon rich nitride, which is generally characterized by a refractive index <2.2^23^,^24^ and according to Miller’s rule, concomitantly lower nonlinear refractive index.

In this paper, we study ultra – silicon rich nitride (USRN) waveguides for their ability to acquire nonlinear phase using ultra – short lengths. The USRN material^25^,^26^ is distinguished from the typical silicon rich nitride platform as it is characterized by a much larger linear refractive index (*n* = 3.1), much larger nonlinear parameters (∼550 W^−1^/m vs. a few W^−1^/m) though both have a sufficiently large band gap to eliminate TPA at the 1.55 μm wavelength. The ultrafast spectral characteristics of USRN waveguide structures are investigated. Using the broad spectrum generated with the short waveguide lengths, quality – factors (Q-factors) of a ring resonator are characterized over a wavelength range of almost 200 nm, outside of that typically available using an erbium – based amplified spontaneous emission source. The output spectra at pass ports of USRN ring resonators using a femtosecond fiber source are examined to understand how the resonator Q-factors and spectral bandwidth change. In addition, the short waveguides with a 1-mm-length scale are sufficiently short to be well below the dispersion length and therefore, nonlinear dynamics are predominantly governed by the waveguide’s nonlinearity.

## Results

Our USRN film is composed of approximately 2:1 of Si:N ratio that is much more silicon rich than stoichiometric silicon nitride (Si:N = 3:4). USRN waveguides which are 600 nm wide and 300 nm thick possess anomalous waveguide dispersion of around 200 ps/nm/km at 1.55 μm. Waveguides used in this experiment have a length of ∼1 mm. Using the previously measured *n*_*2*_ value of the film^25^, the nonlinear parameter for this waveguide is calculated to be ∼550 W^−1^/m. This implies a dispersive length (*L*_D_) of 32 cm and a nonlinear length (*L*_NL_) of <0.19 mm for peak powers >10 W. Given that the *L*_D_ is much longer and the *L*_NL_ is much shorter than the physical waveguide length, the pulse dynamics will be dominated by the nonlinearity rather than dispersion in the waveguide. The large energy bandgap (*E*_g_ ∼ 2.1 eV) is also enough to eliminate TPA of the laser centered at 1.55 μm (∼0.8 eV)^16^.

**Figure 1:**
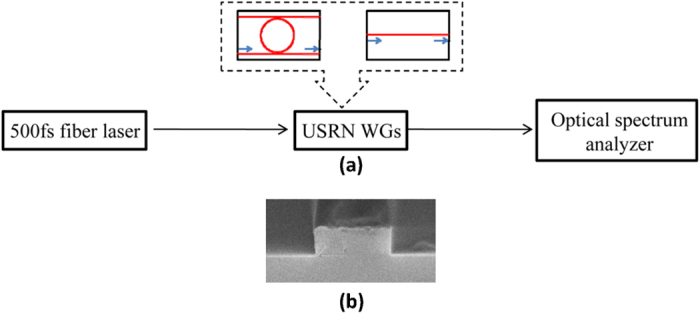
(**a**) Schematic of the experimental setup for measuring fs spectra on USRN waveguides. (**b**) Scanning electron micrograph of a fabricated USRN waveguide.

Figure 1(a) depicts the schematic of the experimental setup to observe output spectra of USRN waveguides. Figure 1(b) shows a scanning electron micrograph of the fabricated USRN waveguide. We use a 500 fs fiber laser centered at 1.55 μm with 20MHz of repetition rate to generate broadened spectra dominated by SPM, which induces temporal variations of the ultrafast pulse intensity. The quasi-TE mode was coupled into the USRN waveguides via tapered lensed fiber. The spectra of output TE signals were observed through optical spectrum analyzer. We use two different structures of USRN waveguides (a bus waveguide with a single ring resonator and a short-length waveguide) to detect the characteristics of their spectral broadening and Q-factor using femtosecond pulses.

**Figure 2:**
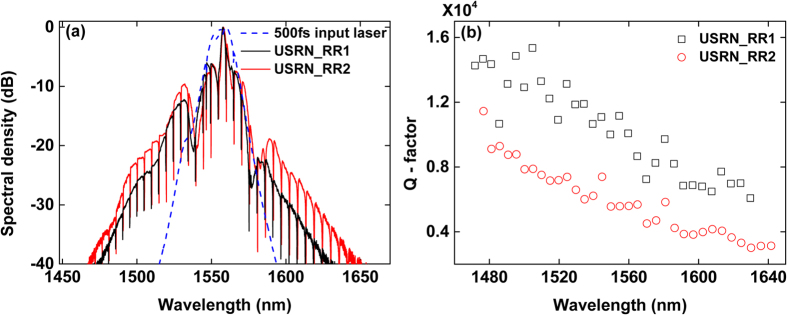
Output fs spectra and Q-factors at pass ports of USRN_RR waveguides. (**a**) Spectral shapes of 500 fs input laser, USRN_RR1 and USRN_RR2. (**b**) Q-factors as a function of central wavelength for USRN_RR1 and RR2.

First, we investigate the output spectra from the pass ports of two USRN add-drop ring resonators (USRN_RR1 and USRN_RR2) waveguides as shown in Fig. 2. USRN_RR1 and USRN_RR2 possessing a 20 μm ring radius both were fabricated to have gaps of 150 nm and 100 nm gap between ring and bus waveguide, respectively. The length of the bus waveguide is 430 μm for USRN_RR1 and 650 μm for USRN_RR2.

The transmission spectra at each pass port are present with the number of resonant peaks as shown in Fig. 2(a). The spectral bandwidth of the input pulse entering the waveguides is measured to be approximately 60 nm at the −30 dB level. The bandwidth measured at the output of USRN_RR1 and RR2 was 110 nm and 130 nm respectively, representing 1.8 and 2.2 times broadening over the original pulse bandwidth. As expected, the output spectral width of USRN_RR2 is a larger because the longer bus waveguide length allows more nonlinear phase to be acquired (Qualitatively, the nonlinear phase acquired in the absence of any nonlinear losses, *φ*_NL_ = γ.*L*_eff_.*P*_peak_, where *L*_eff_ is the effective waveguide length and *P*_peak_ is the peak power used). Two spectral side wings in Fig. 2(a) appear with around 1530 and 1590 nm of center wavelengths. The wings depict an evidence of nonlinear broadening because these are situated outside the region where the fs source possesses spectral content. Consequently, the side wings due to nonlinear spectral broadening facilitate an increase in the number of observable resonance peaks. Each resonance peak is quantified by the Q-factor, defined as center wavelength divided by full-width half maximum (FWHM) bandwidth of a signal (See Fig. 2(b)). The free spectral range (FSR), defined as the spacing in optical frequency between two successive transmission peaks, of two USRN_RR are around 5 nm and the observable resonance peaks span a wavelength range from 1480 to 1640 nm. It is observed that the Q-factor is the highest at the shortest wavelength (USRN_RR1 = 15000 and USRN_RR2 = 11000), and decreases linearly as the wavelength is longer. A greater extent of the optical field is evanescent at longer wavelengths, and consequently, light stored/resonating within the ring couples more easily into the bus waveguide. In line with the definition of Q-factor (=energy stored / energy dissipated per cycle), it follows that Q-factor decreases at longer wavelengths.

**Figure 3:**
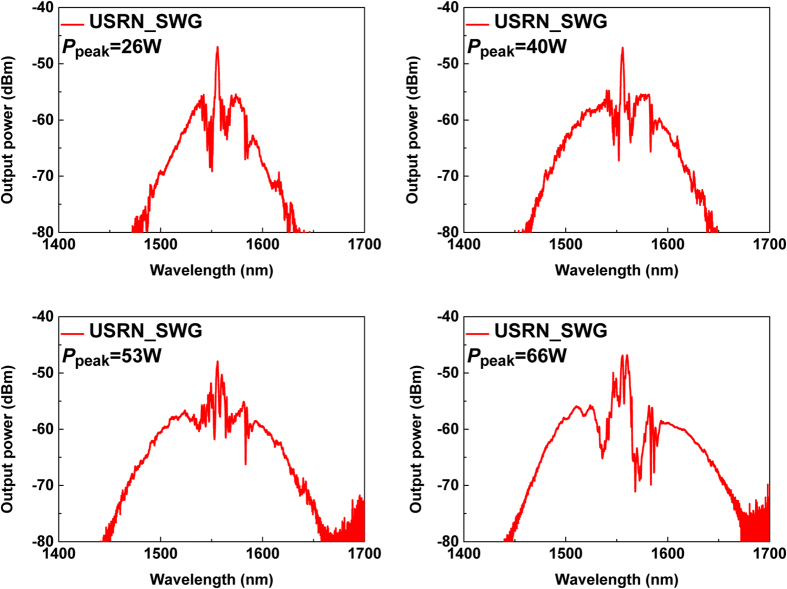
Output spectra of USRN short waveguide (USRN_SWG) with 1.2-mm-length by input peak power.

Next, we investigate the nonlinear spectral broadening properties of a USRN waveguide (USRN_SWG) with 1.2 mm length (∼2× that in the ring resonator with the 650 μm bus waveguide). The output spectra of 500 fs pulses launched into USRN_SWG are observed as the input peak power (*P*_peak_) is increased and the results are shown in Fig. 3. The output spectrum is observed to broaden as the *P*_peak_ is increased from 26 W to 66 W. *P*_peak_ denoted in the figure depicts the coupled input peak power compensated for waveguide loss (10 dB/cm) and output fiber-waveguide coupling loss (∼10 dB per facet). The spectra corresponding with *P*_peak_ = 26 W, 40 W and 53 W are observed to be highly symmetric, therefore SPM-induced broadening is likely to be the dominant effect. Soliton effects and Cherenkov radiation for example is unlikely to be present at these length scales – It often facilitates the broadening of pulse spectra in the regime of supercontinuum, through dispersive wave formation and consequently results in spectra which are asymmetric. At *P*_peak_ = 66 W, the spectral envelope appears slightly asymmetric with different power levels between two wings located on either side of a 1.55-μm spectral peak. The *L*_NL_ calculated for *P*_peak_ = 66 W is ∼3.0 × 10^−2^ mm. The parameter *N*^2^ (=*L*_D_/*L*_NL_) is around 10000, in other words, *N*^2^ ≫ 1. Therefore, it shows that SPM dominates over group velocity dispersion (GVD) for spectral broadening^27^. If we don’t take into account dispersion effect due to dominantly large SPM effect, the asymmetric shapes can arise from the interplay of SPM and high order nonlinear effects. For ultrashort input pulses, self-steepening effect resulting from the intensity dependence of the group velocity occurs and it leads to an asymmetry in the SPM-broadened spectra^28^. Especially, self-steepening at the trailing edge of the pulse produces optical shock formation^29^. When loss is assumed to be zero for simplicity, the shock distance *z*_s_ defined as ∼0.43*L*_NL_/*s* for hyperbolic secant pulse typical in a fiber laser (Where parameter s = 1/*ω*_0_*T*_0,_ ω_0_ = angular frequency, *T*_0_ = *T*_FWHM_/1.76 for hyperbolic secant pulse, *T*_FWHM_ = 500 fs)^25^. The shock distance, *z*_s_ at *P*_peak_ = 66W is around 4 mm at 1.55 μm, respectively. *z*_s_ has same order of length with the waveguide length (=1.2 mm), so self-steepening -induced asymmetric spectral shape might account for the slight asymmetry in the spectrally broadened pulse (see Fig. 3). In addition, SPM-broadening can be limited by the extent of propagation loss that is related with *L*_eff_^27^. The experimentally measured loss coefficient α_wg_ is 2.30 cm^−1^. *L*_eff_ defined as (1 − exp(−α_wg_*L*))/α_wg_ is calculated to be 0.10 cm at a physical length *L* = 1.2 mm. The maximum achievable *L*_eff_ (*L*_eff(max)_) becomes 0.43 cm as *L* goes to infinity. The calculation shows that reducing the propagation loss to 1 dB/cm enables 10% increase in SPM-broadening parameter, *φ*_NL_ owing to the increase of *L*_eff_ value for same waveguide length and experimental condition.

**Figure 4:**
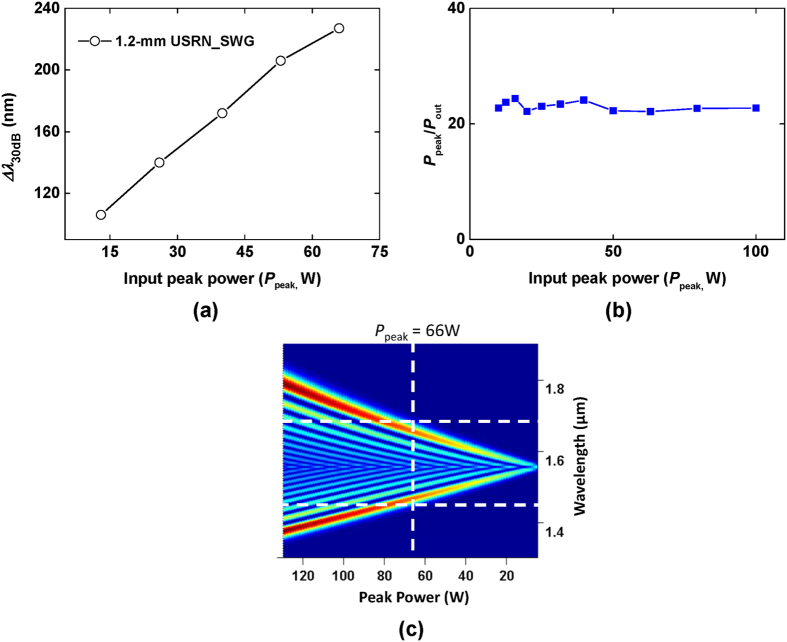
(**a**) Spectral bandwidths of 1.2 mm USRN_SWG at −30 dB level as a function of input peak power (**b**) Measured value of *P*_peak_/*P*_out_ vs. *P*_peak_. The flat profile obtained for *P*_peak_/*P*_out_ as *P*_peak_ is varied implies negligible nonlinear losses. (**c**) Evolution of the 500 fs pulses as a function of the input peak power.

The spectral bandwidth at −30 dB level, Δ*λ*_30 dB_, is also drawn as a function of input peak power in Fig. 4(a). The bandwidth increases linearly up to *P*_peak_ ∼ 66 W with 2.3 nm/W of a slope. To investigate nonlinear loss including TPA effects on the USRN_SWG, output peak power (*P*_out_) is measured as a function of *P*_peak_ up to 100 W. *P*_peak_ varies by using a digital variable attenuator and *P*_out_ is calculated from measured average output power at output tapered fiber via the USRN waveguide. Variation of the reciprocal transmission as the input peak power is varied (*P*_peak_/*P*_out_ versus *P*_peak_^30^) allows us to extrapolate the presence of two photon absorption. *P*_peak_/*P*_out_ is observed in Fig. 4(b) to be almost flat with a standard deviation of 0.78 and an average of 23, implying the absence of nonlinear loss at these power levels. Therefore, the linear increase in both the output bandwidth and power versus *P*_peak_ indicates that USRN waveguide has relatively low nonlinear losses including TPA and free carrier effects up to *P*_peak_ = 100 W. The spectral evolution of 500 fs pulses as a function of input peak power calculated using the Nonlinear Schrödinger equation is shown to increase linearly as *P*_peak_ increases (see Fig. 4(c)). The calculation also reveals that the pulse spectrum spans from 1450–1700 nm at *P*_peak_ ∼ 66 W, which corresponds well with the experimental result (See Fig. 3).

**Figure 5:**
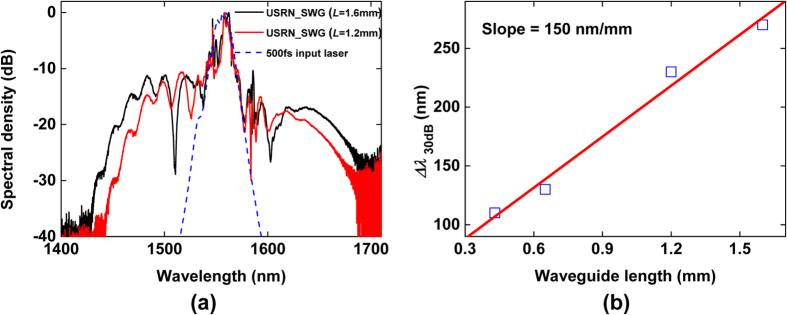
(**a**) Output spectra of 1.2 and 1.6-mm USRN_SWG compared with fs laser spectrum and (**b**) Spectral bandwidth at −30 dB level as a function of waveguide length.

To see the effect of the spectral broadening on the waveguide length, we compared the output spectra as a function of waveguide length as shown in Fig. 5. Figure 5(a) shows measured spectra of 1.2-mm and 1.6-mm length USRN_SWG. Δλ_30 dB_ of 1.2 and 1.6-mm USRN_SWG is around 230 and 270 nm, respectively which is 3.7 and 4.6 times larger than that of the fundamental input. The average measured output power was of 84 μW and 88 μW for 1.2-mm and 1.6-mm USRN_SWG, respectively. Δ*λ*_30 dB_ is also compared for USRN_RR1, USRN_RR2, USRN_SWG (1.2-mm-length) and USRN_SWG (1.6-mm-length). These 4 waveguides have different interaction lengths, so Δ*λ*_30 dB_ in terms of waveguide length can be plotted as shown in Fig. 5(b). If we adopt a linear fit into the graph, the slope is derived to around 150 nm/mm, implying that the bandwidth broadens by 150 nm per millimeter of USRN waveguide.

We define a length-dependent broadening factor, *F*_b_ (=Δ*λ*_out_ /(Δ*λ*_in_ · *L*), mm^−1^) which takes into account the bandwidth ratio between input (Δ*λ*_in_) and output pulses (Δ*λ*_out_) per unit length in the waveguide (*L*). For USRN waveguides with lengths of 1.2 mm and 1.6 mm, the *F*_b_ is 3.19 and 2.81 respectively. The comparison of *F*_b_ between our USRN_SWG with other platforms measured at the telecommunication bands is listed in Table 1. The table includes other reports on both SPM and SCG spectral broadening with a few to hundreds mm of waveguide length. SPM-broadening is normally implemented with hundreds and thousands femtoseconds of pulse widths. *F*_b_ is shown less than 0.1 excluding the silicon nitride waveguide (*F*_b_ ∼ 0.36)^15^ that is achieved due to relative short waveguide length. It indicates that the *F*_b_ value on our waveguide is more than 30 times larger than other SPM-broadened data even though the broadened spectra from our device is also dominated by SPM. Our USRN_SWGs have relatively the shortest length, but the highest *F*_b_ (∼3) among SPM-induced waveguides. This is because γ in our demonstrated USRN waveguides is ~ 550 W^-1^/m^25^ which is large enough to induce spectral broadening even with lengths as short as 1 mm. Comparing the reports on SCG broadening listed in Table 1, *F*_b_ is observed to be ≤1 except for that on ref. 42. We note further that in ref. 42, the longer length scales enable nonlinear effects in addition to pure self – phase modulation such as dispersive wave formation to help facilitate the spectral broadening. The Si_3_N_4_ waveguide (ref. 42) depicts higher *F*_b_ than other SCG-generated waveguides owing to engineering of two zero-GVD points and very small propagation losses (=0.7 dB/cm) for coherent SCG. Despite these benefits to obtain the broad SCG spectrum, however, *F*_b_ is ∼0.7 times smaller than those in our USRN-SWG. Furthermore, we only estimated the minimum (transform-limited) input spectral width at −30 dB level on the Si_3_N_4_ waveguide by time-bandwidth product assuming Sech^2^ pulse, so *F*_b_ value might in fact be smaller than what we expected. Frequency comb generation in a silicon nanophotonic wire waveguide reported in ref. 43 is characterized by an *F*_b_ ∼ 0.24 because of the broad input spectral width even with an octave spanning. Based on Table 1, therefore, our USRN_SWG achieves high spectra-broadened efficiency even with ultra-short lengths because of a combination of large nonlinearity and negligible nonlinear losses. In addition, high powers can be used without TPA effects. Such TPA effects have been widely documented to occur at sub – watt powers in silicon waveguides.

Using the USRN waveguides with a length of 1 mm, an output bandwidth of around 200 nm centered at 1550 nm is achieved.

## Discussions

When we consider the third order dispersion (TOD), TOD length, defined as *L*_D_′ = *T*_0_^3^/*β*_3_, is ∼0.3 m (*β*_3_ = 68 ps^3^/km at 1.555 μm)^25^. *L*_D_ and *L*_D_′ are about 0.3 m–4 orders of magnitude larger magnitude than *L*_NL_ and 300 times larger than the waveguide length in USRN_SWG (∼1 mm). Consequently, we don’t take into account dispersion effects in the broadened spectra. The asymmetric spectral shape as shown in Fig. 3 can be attributed to other nonlinear effects such as modulation instability as well as self-steepening effect because the waveguide’s anomalous dispersion could trigger several nonlinear processes for extending the spectra. The spectral shape around 1.55-μm-wavelength doesn’t show significant phase shift as input peak power increase, but instead, the spectrum broadens with high order sidebands as shown in Fig. 3. Therefore, it is difficult to identify the nonlinear effects present by solely observing output spectral shapes. Through observations of the broadened spectra, their evolution and the length scales involved, self – phase modulation is likely to be the dominant effect contributing to the spectral broadening.

## Conclusions

We have studied ultrafast wideband broadening of USRN waveguides which are around 1 mm in length. USRN add-drop ring resonators broaden the output spectra by a factor of around 2 compared with the bandwidth of the 500 fs input pulses. The Q-factors, which range from 1480 and 1680 nm, measured at pass ports of the resonators are examined to have the highest values of 11000 and 15000 according to the bus waveguide length with a free spectral range of 5 nm. In addition, short waveguides of 1.2 mm and 1.6 mm USRN_SWG facilitated broadening of the spectral bandwidth to 230 and 270 nm, corresponding to 3.8 and 4.5 times larger than that of the fundamental input, respectively. Owing to the high nonlinear parameter (γ ∼ 550 W^−1^/m) and low TPA of USRN, the waveguides possess a higher *F*_b_ (∼3) than that in other CMOS-compatible platforms. Therefore, we can obtain output bandwidths of 200 nm (0.2 octaves) centered at 1.55 using ultra-short USRN waveguides with 1 mm in length. Consequently, the demonstrated USRN waveguides could be well poised to enable CMOS-compatible nonlinear optical devices with much smaller footprints due to its short length and high nonlinearity, and find applications in wideband and multi-wavelength generation at the telecommunications wavelength.

## Methods

### Measurements

A 500 fs fiber laser centered around 1.55 μm with 20 MHz repetition rate was used as a fundamental source. The laser polarization maintains with quasi TE mode, linearly horizontal-polarized by using in-line fiber polarization controller. The polarization-maintained input light was coupled into USRN waveguides via tapered lensed fiber. Output TE signals were also coupled into the same type of polarization maintaining tapered fiber and their spectra were observed through optical spectrum analyzer.

### Device fabrication

Fabrication of the waveguides was performed by first depositing 300 nm of ultra – silicon rich nitride films using inductively coupled chemical vapor deposition. In order to minimize N – H bonds which are known to cause materials losses close to 1.55 μm, precursor gases used were SiH_4_ and N_2_. The gas flow ratio of SiH_4_:N_2_ used to deposit the films in this work was ∼2:1. The deposition temperature used was 250 °C. The waveguides were then defined using electron – beam lithography followed by reactive ion etching. Finally, 2 μm of SiO_2_ overcladding was deposited using plasma enhanced chemical vapor deposition.

## Additional Information

**How to cite this article**: Choi, J. W. *et al.* Wideband nonlinear spectral broadening in ultra-short ultra - silicon rich nitride waveguides. *Sci. Rep.* **6**, 27120; doi: 10.1038/srep27120 (2016).

## Figures and Tables

**Table 1:** Comparison of broaden factors (*F*_b_) among various CMOS-compatible platforms at telecommunication bands. (*assuming Sech^2^ pulse shape, FCG: Frequency comb generation).

WG Type	Output bandwidth at −30 dB level (Δ*λ*_out_, nm)	Input bandwidth at −30 dB level (Δ*λ*_in_, nm)	WG length (*L*, mm)	Input peak power (*P*_peak_, W)	Input pulse width (fs)	Broaden factor, (*F*_b_=Δ*λ*_out_ /(Δ*λ*_in_·*L*), mm^−1^)
Silicon rib^31^ (SPM)	∼7.3	∼5.3	20	∼110	4000	0.07
Silicon^32^ (SPM)	9.1	3.5	20	85	3000	0.13
Hydex glass^33^ (SPM)	280	∼25	450	240	350	∼0.025
Silicon nitride^15^ (SPM)	∼30	∼14	6	500	7000	∼0.36
Silicon^34^ (SCG)	∼350	>85*	4.7	1	100	∼0.88
SOI^35^ (SCG)	500	>235*	3	25	50	<0.71
Amorphous silicon^36^ (SCG)	∼200	∼12	10	45	1000	∼1.67
Amorphous silicon^37^ (SCG)	∼500	>71*	10	13	180	<0.70
Hydex glass^38^ (SCG)	300 at −20dB level	110 at −20 dB level	450	1450	100	∼0.006
Polycrystalline anatase TiO_2_^39^ (SCG)	190 at −15 dB level	50 at −15 dB level	9	2600	170	0.42
Silicon nitride^40^ (SCG)	∼720	>188*	5.5	661	65	<0.70
Silicon nitride^41^ (SCG)	1360	>42*	43	800	200	<0.75
Silicon nitride^42^ (SCG)	1080	>61*	7.5	402	92	<2.36
Silicon^43^ (FCG)	∼1180	∼500	10	225	70	∼0.24
USRN_SWG (this work)	230	60	1.2	116	500	3.19
USRN_SWG (this work)	270	60	1.6	121	500	2.81
